# Facial profile evaluation and prediction of skeletal class II patients during camouflage extraction treatment: a pilot study

**DOI:** 10.1186/s13005-023-00397-8

**Published:** 2023-12-04

**Authors:** Runzhi Guo, Yuan Tian, Xiaobei Li, Weiran Li, Danqing He, Yannan Sun

**Affiliations:** 1 Department of Orthodontics, Peking University School and Hospital of Stomatology, National Center for Stomatology & National Clinical Research Center for Oral Diseases, 22 Zhongguancun Avenue South, Haidian District, Beijing, 100081 P.R. China; 2 Department of Operational and Development Office, Peking University School and Hospital of Stomatology, Beijing, 100081 P.R. China

**Keywords:** Esthetics, Cephalometry, Visual analog scale, Orthodontic camouflage treatment, Machine-learning

## Abstract

**Background:**

The evaluation of the facial profile of skeletal Class II patients with camouflage treatment is of great importance for patients and orthodontists. The aim of this study is to explore the key factors in evaluating the facial profile esthetics and to predict the posttreatment facial profile esthetics of skeletal Class II extraction patients.

**Methods:**

124 skeletal Class II extraction patients were included. The pretreatment and posttreatment cephalograms were analyzed by a trained expert orthodontist. The facial profile esthetics of pretreatment and posttreatment lateral photographs were evaluated by 10 expert orthodontists using the visual analog scale (VAS). The correlation between subjective facial profile esthetics and objective cephalometric measurements was assessed. Three machine-learning methods were used to predict posttreatment facial profile esthetics.

**Results:**

The distances from lower and upper lip to the E plane and U1-APo showed the stronger correlation with profile esthetics. The changes in lower lip to the E plane and U1-APo during extraction exhibited the stronger correlation with changes in VAS score (r = − 0.551 and r = − 0.469). The random forest prediction model had the lowest mean absolute error and root mean square error, demonstrating a better prediction accuracy and fitting effect. In this model, pretreatment upper lip to E plane, pretreatment Pog-NB and the change of U1-GAll were the most important variables in predicting the posttreatment score of facial profile esthetics.

**Conclusions:**

The maxillary incisor protrusion and lower lip protrusion are key objective indicators for evaluating and predicting facial profile esthetics of skeletal Class II extraction patients. An artificial intelligence prediction model could be a new method for predicting the posttreatment esthetics of facial profiles.

**Supplementary Information:**

The online version contains supplementary material available at 10.1186/s13005-023-00397-8.

## Background

Skeletal Class II malocclusion is frequently characterized by a convex facial profile, which is caused by mandibular retrusion, maxillary protrusion, or a combination of both [1]. Esthetic concerns are the major motivating factor of patients seeking orthodontic treatment for this condition; hence, an important treatment goal is to improve their facial appearance [2, 3]. Although orthodontic-orthognathic treatment could address the skeletal discrepancy and significantly improve the facial profile, it may be difficult to convince patients to have orthognathic surgery [4]. In such circumstances, camouflage orthodontic therapy is an alternative treatment; it consists of extraction of premolars to retract the anterior teeth. With camouflage orthodontic treatment, a relatively pleasing profile can be achieved [5, 6]. However, if the skeletal discrepancy is severe or the extent of anterior teeth retraction is excessive, this treatment could lead to an undesirable dished-in profile for skeletal Class II patients [7]. Czarnecki et al. reported that the retrusive profile was least acceptable; 62% of participants rated it the worst [8]. Therefore, the evaluation of the facial profile of skeletal Class II patients with camouflage treatment is of great importance for patients and orthodontists.

Lateral cephalometric radiographs and facial photography play an important role in orthodontists’ evaluation of facial attractiveness. Cephalometry analysis can objectively reflect the relationship between the surface soft tissue and underlying skeletal and dental structure. Almost all cephalometric analyses include some measurements that can be used to evaluate facial attractiveness [9–11]. Some previous studies have reported correlations between cephalometric measurements and facial attractiveness, and they found that some cephalometric measurements could be selected as reliable facial attractiveness indices [12, 13]. The esthetics of facial profiles are abstract and subjective. Judgments of profile esthetics from lateral photography based on visual analog scale (VAS) are commonly used in the orthodontic field, which has been proved to be reliable and valid [14]. Previous studies have explored the correlation between cephalometric measurements and VAS assessment of profile esthetics in bimaxillary protrusion patients [15, 16]. For skeletal Class II patients, the contribution of these objective measurements to subjective assessment of profile esthetics has not been established. Exploring the key cephalometric measurement related to subjective facial profile evaluation of skeletal Class II patients is essential in treatment planning.

Considering the high esthetic need of skeletal Class II patients, prediction of posttreatment profile esthetics after camouflage extraction is essential in treatment planning. If the predicted score of posttreatment profile esthetics is not satisfied, orthodontic-orthognathic treatment may be needed. Prediction of profile esthetic is complicated and depends on many factors [17]. For this reason, no reliable prediction model has been previously reported. Machine learning is increasingly used in diagnosis and prognosis of diseases [18–20]. Compared to conventional prediction models, machine learning can develop supervised algorithms capable of incorporating many variables, which makes it accurate and practical for disease prediction. To date, the machine-learning method has not been applied to predict profile esthetic change after camouflage orthodontic treatment.

The purpose of this study is to evaluate the correlation between objective cephalometric measurements and subjective assessment of profile esthetics in skeletal Class II extraction patients, and further predict the posttreatment profile esthetics using machine-learning methods. The null hypotheses are as follows: (1) there is a strong correlation between some objective cephalometric measurements and subjective assessment of profile esthetics in skeletal Class II extraction patients; and (2) the machine learning prediction of posttreatment profile esthetics of skeletal Class II extraction patients is achievable and reliable.

## Methods

### Participants

Our retrospective study protocol was approved by the Peking University School and Hospital of Stomatology Ethics Committee (PKUSSIRB-202,168,141). Patients who had completed orthodontic extraction treatment at the Department of Orthodontics, Peking University School and Hospital of Stomatology between June 2015 and June 2022 were enrolled and further included if they met the following criteria: 12–30 years old, skeletal Class II (ANB angle ≥ 5º), mild crowding in upper and lower dentition (i.e., < 3 mm), the most anterior points of upper or lower lips in advance of E line, a treatment plan that involved extraction of four premolars and retraction of anterior teeth, no cleft lip and/or palate, no craniofacial syndromes, no orthognathic surgery, and no cosmetic facial surgery. All patients were treated with pre-adjusted MBT appliances (Shinye, Hangzhou, China). In order to analyze the effect of anterior teeth retraction on facial profile esthetics, the amount of anterior teeth retraction is not limited. The mini-screws could be used to retract anterior teeth if the maximum anchorage is required. Ultimately, 124 patients (mean age: 18.87 ± 5.35 years) were included: 76 female patients and 48 male patients. The treatment duration of included patients was 2.60 ± 1.80 years. Considering the facial growth in adolescents, the patients were further divided into an adolescent group (age < 18 years) and an adult group (age ≥ 18 years). All patients provided written informed consent to participate.

### Measurements

#### Objective measurements of soft tissue, skeletal tissue, and incisor position

**Fig. 1:**
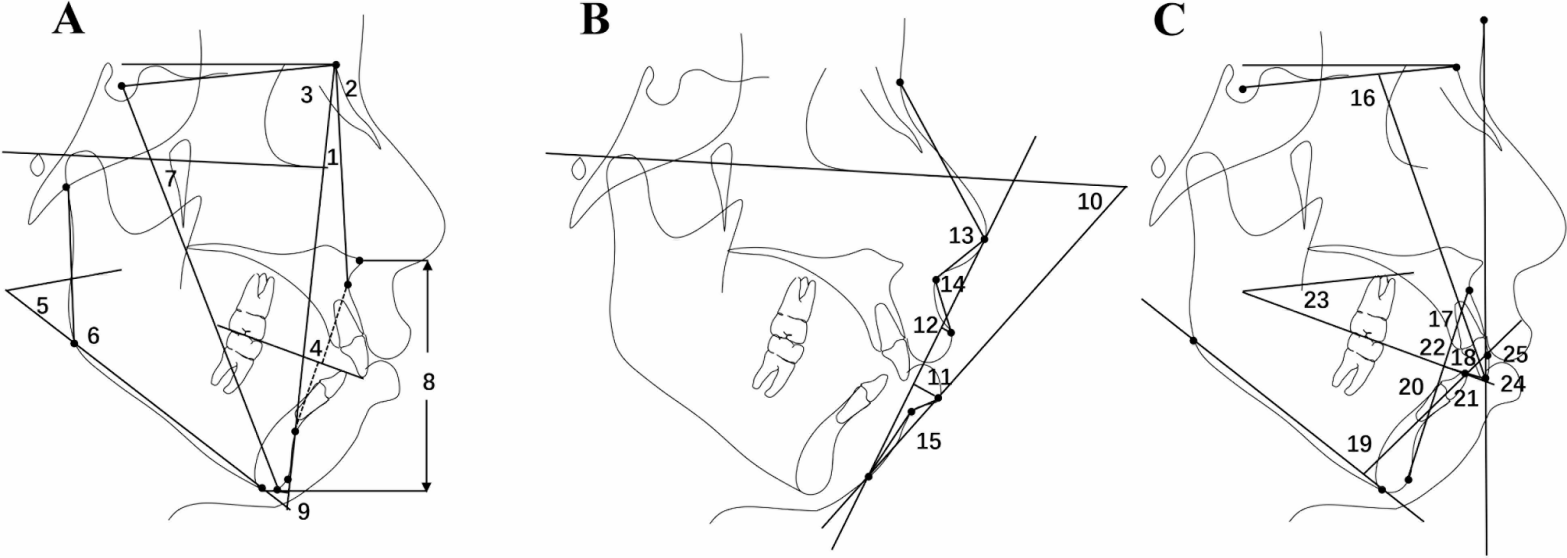
The cephalometric measurements. (**A**) skeletal measurements: 1, ANB; 2, SNA; 3, SNB; 4, Wits Appraisal; 5, MP-SN; 6, Ar-Go-Me; 7, Y Axis; 8, Lower facial height; 9, Pog-NB. (**B**) soft tissue measurements: 10, Z Angle; 11, Lower Lip to E Plane; 12, Upper Lip to E Plane; 13, Nose Prominence; 14, Nasolabial Angle; 15, Mentolabial Angle. (**C**) dental measurements: 16, U1-SN; 17, U1-APo angle; 18, U1-APo distance; 19, L1-MP; 20, L1-APo angle; 21, L1-APo distance; 22, U1-L1; 23, Occlusal Plane to SN; 24, U1-GALL; 25, FA-GALL

According to ALARA (as low as reasonably achievable), the cephalograms of all included patients were obtained before orthodontic appliance placement (T1) and after orthodontic appliance removal (T2). Pretreatment and posttreatment cephalograms were digitally analyzed by a trained expert orthodontist using Dolphin Imaging Software (version 11.7, Dolphin Imaging System, Canoga Park, CA, USA). Before the data were analyzed, the magnification differences between two cephalograms were corrected and calibrated. The head position of the cephalogram was reoriented 7 degrees inferior to the SN plane with Sella registration. As shown in Fig.1, 25 measurements, consisting of 9 skeletal measurements, 6 soft tissue measurements, and 10 dental measurements, were selected and analyzed in this study. The definitions of the included measurements were listed in Supplementary Table 1.

#### Subjective evaluation of profile esthetics

Pretreatment and posttreatment lateral images were obtained in the natural head position with forehead exposure and lips in the rest position. Facial esthetics were evaluated by 10 expert orthodontists with more than 10 years’ experience (5 men and 5 women, age 35–50 years). As described in a previous study [15], the pretreatment and posttreatment lateral photographs of the patients were randomly presented in a slide show and evaluated using VAS from 0 (very unpleasing) to 100 (very pleasing). The final score was the average of 10 scores.

#### Methods of predicting posttreatment profile esthetics

Due to the high correlation between independent variables, the multivariate linear regression model is prone to instability. Thus, we established three machine-learning models, including multiple stepwise regression (MSR), support vector machine (SVM) and random forest (RF), which were commonly used for regression prediction, to predict and analyze the posttreatment facial profile esthetics of skeletal Class II patients. The following variables were selected as input variables: 16 pretreatment cephalometric measurements that were significantly correlated with pretreatment VAS score based on Pearson correlation results, 3 incisor position changes (ΔU1-SN, ΔL1-MP, and ΔU1-GALL) that could be planned before treatment, as well as the age of the patients (numerical variable). The posttreatment VAS score was the output variable. A random number generator was used to divide the samples into 75% training set (93 patients) and 25% testing set (31 patients). By tuning the parameters, the optimal parameter combination and the final prediction model was obtained. Different machine learning algorithms were used to perform regression training on the samples in the training set. The mean absolute error (MAE) and root mean square error (RMSE) between the predicted VAS score and the actual VAS score were used to evaluate the fitting performance of three models. RMSE refers to the square root of the average square difference between the predicted value and the actual observation, which can measure the deviation between observed value and true value. MAE refers to the average absolute value of the absolute deviation of each measurement value, which can accurately reflect the size of the actual prediction error. The smaller RMSE and MAE values represented the smaller differences between our predicted values and true values, which indicated the higher accuracy of the model. All analyses were performed using Stata version 17.0 (StataCorps; College Station, Texas).

### Statistical analysis

The statistical analyses were performed using SPSS software (version 26; SPSS, Chicago, IL, USA). To assess reliability, 20 patients were randomly selected. The pretreatment and posttreatment cephalograms of these patients were reanalyzed by the same orthodontist after an interval of 2 weeks. The lateral photography was reevaluated by 10 orthodontists a month later. The mean and standard deviation of measurements were calculated. The Shapiro–Wilk test was used to evaluate the normality of measurement distributions. For normally distributed measurements, the t-tests and the independent-samples t-test were used to compare the mean differences between timepoints and age groups, respectively. The Wilcoxon signed-rank test was performed when the measurement distributions were not normally distributed. Pearson correlation analysis was applied to evaluate the correlation between the cephalometric measurements and subjective assessment of profile esthetics. Statistical significance was set at *P* < 0.05.

## Results

### Descriptive information for cephalometric measurements and facial profile esthetics

In all, 124 skeletal Class II extraction patients were included: 67 adults (mean age: 22.90 ± 3.91 years) and 57 adolescents (mean age: 14.14 ± 1.66 years). The pretreatment and posttreatment measurements, including the subjective VAS score and cephalometric measurements, were listed in Table 1. The intraclass correlation coefficients of all cephalometric measurements were 0.875–0.967, indicating good reliability. The intraclass correlation coefficient of the VAS score was 0.916. After four premolar extraction, dental, soft tissue, and some skeletal measurements were significantly different. The Z angle and the nasolabial angle were significantly greater, and the ANB angle was significantly smaller (*P* < 0.001). The VAS score significantly increased from 65.89 ± 10.09 at T0 to 77.60 ± 5.23 at T1. As shown in Supplementary Table 2, the difference in the change in VAS score between adult and adolescent patients was not significant. The adolescent group showed a greater increase in SNB angle, lower facial height, and nose prominence than the adult group (*P* < 0.001). These data indicate that the adolescents showed significant mandibular and nose growth during the extraction treatment.

**Table 1:** Pretreatment and posttreatment subjective VAS scores and objective measurements of skeletal Class II patients

Variable	T0			T1			*P*
	Mean	SD		Mean	SD		
**VAS score**	65.885	10.086		77.598	5.228		< 0.001^**^
**Skeletal measurements**							
ANB (º)	6.652	1.242		5.348	1.454		< 0.001^**^
SNA (º)	83.793	3.666		82.544	3.510		< 0.001^**^
SNB (º)	77.144	3.393		77.193	3.425		0.502
Wits Appraisal (mm)	3.839	2.068		0.677	1.787		< 0.001^**^
MP-SN (º)	38.644	5.521		39.035	5.551		0.001^*^
Ar-Go-Me (º)	124.002	6.871		124.444	6.873		< 0.001^**^
Y Axis (º)	73.380	3.458		73.454	3.507		0.298
Lower facial height (%)	55.752	1.879		56.082	1.862		< 0.001^**^
Pog-NB (mm)	-0.365	1.745		0.028	1.767		< 0.001^**^
**Soft tissue measurements**							
Z Angle (º)	59.967	7.618		69.406	8.729		< 0.001^**^
Lower Lip to E Plane (mm)	4.298	2.439		0.822	1.830		< 0.001^**^
Upper Lip to E Plane (mm)	0.693	1.966		-0.987	1.812		< 0.001^**^
Nose Prominence (º)	18.512	2.030		18.744	2.192		0.027^*^
Nasolabial Angle (º)	105.539	10.775		110.073	10.626		< 0.001^**^
Mentolabial Angle (º)	128.131	42.072		137.652	14.116		< 0.001^**^
**Dental measurements**							
U1-SN (º)	105.757	8.349		96.743	6.695		< 0.001^**^
U1-APo (º)	36.994	7.693		25.902	5.036		< 0.001^**^
U1-APo (mm)	9.686	2.332		5.363	1.537		< 0.001^**^
L1-MP (º)	96.828	6.908		91.685	6.301		< 0.001^**^
L1-APo (º)	24.236	5.556		21.564	4.584		< 0.001^**^
L1-APo (mm)	4.610	2.443		2.422	1.601		< 0.001^**^
U1-L1 (º)	118.760	11.160		132.531	7.716		< 0.001^**^
Occlusal Plane to SN (º)	18.106	4.026		20.949	4.173		< 0.001^**^
U1-GALL (mm)	2.045	3.664		-2.839	3.365		< 0.001^**^
FA-GALL (mm)	2.446	3.369		-1.713	3.122		< 0.001^**^

^*^P < 0.05, ^**^P < 0.001

### Correlation between pretreatment cephalometric measurements and facial profile esthetics

The Pearson correlation between the pretreatment VAS scores and pretreatment cephalometric measurements was shown in Table 2. The measurements were listed in descending order of the absolute value of the correlation coefficient. In all, 16 cephalometric measurements were significantly correlated with the VAS score of profile esthetics. Among these measurements, the distance from the lower lip to the E plane showed the strongest correlations with profile esthetics, with a correlation coefficient of − 0.580 (*P* < 0.001). The distance from the upper lip to the E plane (r = − 0.482, *P* < 0.001), U1-APo distance (r = − 0.477, *P* < 0.001), and L1-APo distance (r = − 0.474, *P* < 0.001) were moderately correlated with the VAS score. As for skeletal measurements, ANB angle was negatively correlated with VAS score (r = − 0.312), with *P* values less than 0.001. The vertical skeletal measurements, such as MP-SN angle, lower facial height, and Ar-Go-Me angle, were not significantly correlated with VAS score. The Pog-NB distance, which reflected the chin morphology, was positively correlated with VAS score (r = 0.402, *P* < 0.001). We further explored the correlations between profile esthetics and cephalometric measurements in adult and adolescent skeletal Class II patients. As shown in Supplementary Table 3, there were 14 cephalometric measurements in the adult group and 10 in the adolescent group that were significantly correlated with VAS score. In the adult group, the lip and upper incisor protrusion measurements, including lower lip to E plane (r = − 0.640, *P* < 0.001), upper lip to E plane (r = − 0.593, *P* < 0.001), U1-APo distance (r = − 0.505, *P* < 0.001) and U1-APo angle (r = − 0.487, *P* < 0.001), showed strong correlations with VAS score. A stronger correlation was found between lower incisor position (L1-APo distance, L1-MP angle, and L1-APo angle) and VAS scores in the adolescent group (r = − 0.478, *P* < 0.001; r = − 0.467, *P* < 0.001; r = − 0.443, *P* < 0.001).

**Table 2:** Pearson correlation between pretreatment subjective VAS scores and objective measurements in 124 skeletal Class II patients

Variable	r	*P*	order
Lower Lip to E Plane (mm)	-0.580	< 0.001^**^	1
Upper Lip to E Plane (mm)	-0.482	< 0.001^**^	2
U1-APo (mm)	-0.477	< 0.001^**^	3
L1-APo (mm)	-0.474	< 0.001^**^	4
Z Angle (º)	0.445	< 0.001^**^	5
U1-L1 (º)	0.413	< 0.001^**^	6
Pog-NB (mm)	0.402	< 0.001^**^	7
U1-APo (º)	-0.402	< 0.001^**^	8
U1-GALL (mm)	-0.340	< 0.001^**^	9
FA-GALL (mm)	-0.318	< 0.001^**^	10
ANB (º)	-0.312	< 0.001^**^	11
L1-MP (º)	-0.307	0.001^*^	12
L1-APo (º)	-0.273	0.002^*^	13
U1-SN (º)	-0.240	0.007^*^	14
Wits Appraisal (mm)	-0.188	0.037^*^	15
Nasolabial Angle (º)	0.180	0.046^*^	16
SNA (º)	-0.142	0.115	17
Lower facial height (%)	-0.101	0.264	18
MP-SN (º)	-0.086	0.344	19
Y Axis (º)	-0.075	0.405	20
Ar-Go-Me (º)	-0.072	0.428	21
Mentolabial Angle (º)	0.070	0.440	22
SNB (º)	-0.040	0.659	23
Nose Prominence (º)	-0.012	0.899	24
Occlusal Plane to SN (º)	-0.006	0.943	25

^*^P < 0.05, ^**^P < 0.001

### Correlation between changes in cephalometric measurements and changes in subjective evaluation of facial profile

As shown in Table 3, the change in distance from the lower lip to the E plane showed the strongest correlation with change in VAS score for profile esthetics (r = − 0.551, *P* < 0.001). The change in the upper lip to the E plane was moderately correlated with the change in VAS scores (r = − 0.255, *P* = 0.004). The ΔU1-APo distance and ΔL1-APo distance were significantly correlated with the ΔVAS scores (r = − 0.469, *P* < 0.001; r = − 0.454, *P* < 0.001). These data indicate that retraction of the lip and incisor may significantly influence the VAS scores of facial profile esthetics. Change in the nasolabial angle was positively correlated with changes in VAS score (r = 0.190, *P* = 0.034). The change in lower facial height was negatively correlated with the VAS score change (r = − 0.213, *P* = 0.017), indicating that an increase of lower facial height in skeletal Class II extraction patient could lead to an undesirable profile. The Pearson correlations between changes in VAS score and changes in cephalometric measurements in adult and adolescents were shown in Supplementary Table 4. The change in the distance from the upper lip to the E plane was significantly correlated with changes in VAS score in adult patients (r = − 0.344, *P* < 0.001) but not in adolescents (r = − 0.177, *P* = 0.187). In adults, ΔU1-APo distance (r = − 0.492, *P* < 0.001) was more correlated with ΔVAS scores than ΔL1-APo distance (r = − 0.421, *P* < 0.001). Meanwhile, ΔL1-APo distance (r = − 0.461, *P* < 0.001) was more correlated with ΔVAS scores than ΔU1-APo distance (r = − 0.395, *P* < 0.001) in the adolescent group.

**Table 3:** Pearson correlation between subjective VAS score change and objective measurement change in skeletal Class II extraction patients

Variable	r	*P*	order
Lower Lip to E Plane (mm)	-0.551	< 0.001^**^	1
U1-APo (mm)	-0.469	< 0.001^**^	2
L1-APo (mm)	-0.454	< 0.001^**^	3
U1-L1 (º)	0.445	< 0.001^**^	4
Z Angle (º)	0.389	< 0.001^**^	5
L1-MP (º)	-0.381	< 0.001^**^	6
U1-APo (º)	-0.363	< 0.001^**^	7
FA-GALL (mm)	-0.360	< 0.001^**^	8
L1-APo (º)	-0.355	< 0.001^**^	9
U1-SN (º)	-0.329	< 0.001^**^	10
U1-GALL (mm)	-0.279	0.002^*^	11
Upper Lip to E Plane (mm)	-0.255	0.004^*^	12
Lower facial height (%)	-0.213	0.017^*^	13
Nasolabial Angle (º)	0.190	0.034^*^	14
MP-SN (º)	-0.171	0.058	15
Ar-Go-Me (º)	-0.162	0.073	16
SNA (º)	-0.140	0.122	17
ANB (º)	-0.135	0.134	18
Y Axis (º)	-0.105	0.244	19
Wits Appraisal (mm)	-0.069	0.445	20
Pog-NB (mm)	0.063	0.490	21
Mentolabial Angle (º)	0.058	0.520	22
Occlusal Plane to SN (º)	-0.039	0.667	23
SNB (º)	0.012	0.896	24
Nose Prominence (º)	0.009	0.921	25

^*^P < 0.05, ^**^P < 0.001

### Prediction of posttreatment profile esthetics after camouflage extraction in skeletal class II patients

**Fig. 2:**
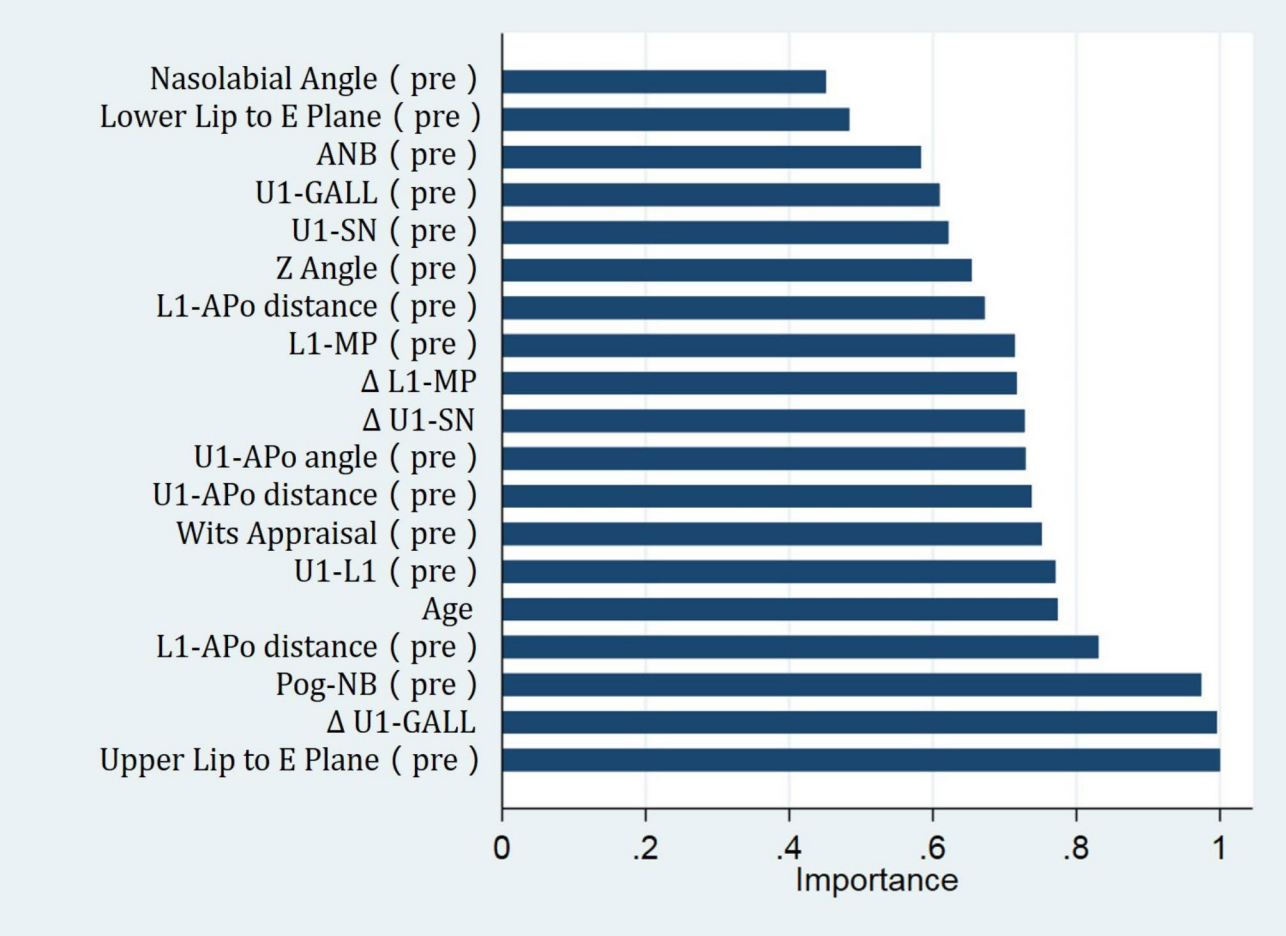
The importance of predictor variables in RF model

**Fig. 3:**
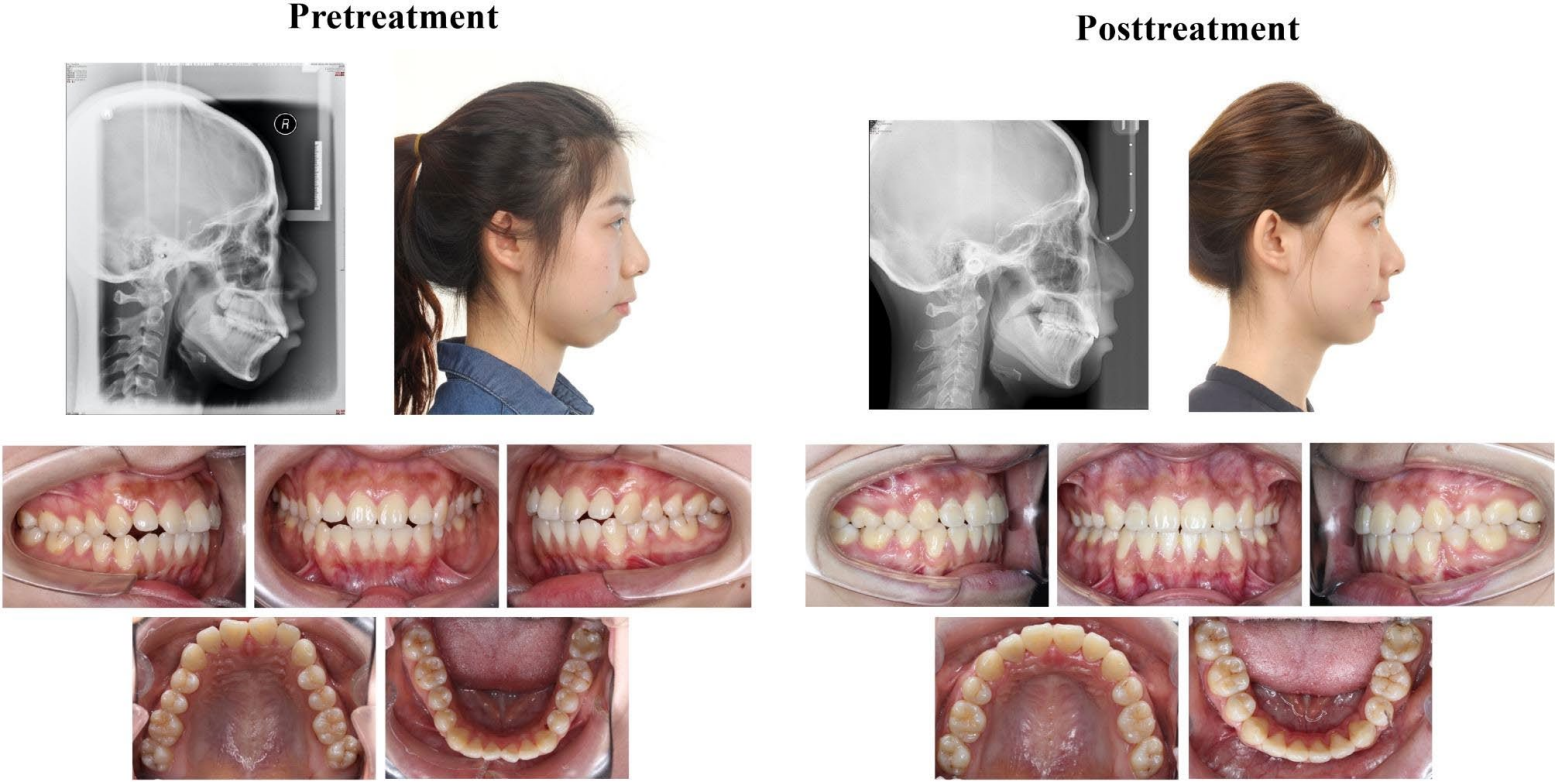
The illustration of camouflage orthodontic treatment in a skeletal class II patient

Based on the results shown in Table 2, 16 of 25 cephalometric measurements at T0 that were significantly correlated with facial profile esthetics were selected as input variables. Age was also selected as an input variable (considering the different correlations between cephalometric measurements and facial profile esthetics in adult and adolescent patients), as well as three incisor position changes, the ΔU1-SN, ΔL1-MP, and ΔU1-GALL (which should be planned as treatment goals). Hence, the posttreatment VAS score was predicted based on age, 16 pretreatment objective measurements, and 3 incisor position changes using three machine-learning models. 75% of the samples were utilized as the training set and 25% of the samples were utilized as the testing set for cross validation to ensure the stability of the model. The hyperparameter of random forest model was adjusted to minimize the out-of-bag (OOB) error, mainly adjusting the number of iterations and variables. When the iteration was 500 and variable was 9, the OOB error was minimized. RMSE and MAE, the important indicators for evaluating model performance in machine learning, were evaluated in different models (Table 4). The smaller RMSE and MAE values represented the smaller difference between the predicted and true values, which indicated higher accuracy of the model. Compared to other prediction models, RF had the lowest RMSE (3.106) and MAE (3.930), demonstrating a better prediction accuracy and fitting effect. Thus, RF had the best performance. The variable importance in the RF prediction model was shown in Fig. 2. The pretreatment upper lip protrusion (upper lip to E plane), pretreatment chin morphology (Pog-NB) and upper incisor retraction (ΔU1-GAll) were the most important variables in predicting scores for facial profile esthetics. For instance, the pretreatment and posttreatment average VAS scores of facial profile of the patient were 48.1 and 75.8, respectively (Fig. 3), and the predicted VAS score of posttreatment facial profile using the RF model was 77.1, indicating the RF model was relatively reliable and accurate.

**Table 4:** Comparison of three model prediction performance

Model	MAE	RMSE
MSR	4.664	5.680
SVM	4.045	4.947
RF	3.106	3.930

MAE: mean absolute error; RMSE: root mean square error; MSR: multiple stepwise regression; SVM: support vector machine; RF: random forest

## Discussion

Previous studies have mainly focused on evaluating the soft tissue changes of skeletal Class I and Class II extraction patients [21, 22]. In this study, the correlation between subjective facial profile esthetics and objective cephalometric measurements in skeletal Class II patients, and the correlation between facial profile changes and cephalometric measurement changes during camouflage extraction treatment, were first assessed. We found a strong association between objective measurements and subjective assessment of profile esthetics, and we used three machine-learning models to predict posttreatment profile esthetics.

Compared to skeletal Class I patients, skeletal Class II patients often have associated facial deformity, which can affect the facial profile. Which cephalometric measurement is most related to facial profile esthetics in skeletal Class II patients? To date, there have been no studies to answer this question. In this study, lip protrusion and incisor position, particularly lower lip protrusion, were first found to be a critical factor in evaluating the facial profile esthetics. In our previous study, the distance between facial-axis point (FA) of maxillary incisor and G line (GALL), which was proposed by Andrews et al., was more convenient and stable for evaluating facial profile esthetics in skeletal Class I patients [23]. While, to evaluate the facial profile esthetics of skeletal Class II patients, FA-GALL is not as sensitive as U1-APo distance, which considered both maxillary incisor position and maxillary bone position. Hence, the FA-GALL and U1-APo distance are recommended to evaluate facial profile esthetics in skeletal Class I and Class II patients, separately. Interestingly, the maxillary incisor position (U1-APo distance) showed a stronger correlation with facial profile esthetics in adult patients, whereas the mandibular incisor position (L1-APo distance) was more sensitive in adolescent patients. In addition, the chin morphology (Pog-NB distance) showed a positive correlation with facial profile esthetics. Our findings are consistent with Huang et al., who found that lip protrusion, incisor position, and chin morphology are the key measurements correlated with the profile esthetics of bimaxillary protrusion patients (ANB angle: 4.76 ± 1.91) [15]. Hence, a harmonious lower third of the face plays an important role in facial profile esthetics. As for the skeletal pattern, the sagittal skeletal patterns (ANB angle) have a significant influence on facial profile evaluation. In our study, the patients mainly had mandibular retrusion (SNB angle: 77.14 ± 3.39); maxillary positions (SNA angle: 83.79 ± 3.67) were relatively normal. This finding is in agreement with the findings of Krooks et al., reporting that sagittal skeletal dimension is the most important factor when evaluating facial profile esthetics [24].

For skeletal Class II patients, camouflage extraction treatment can mask the skeletal deformity through dental compensation and improve the facial profile. However, an unattractive dished-in profile may also occur if the anterior teeth are excessively retracted [7]. In our study, the amount of maxillary anterior teeth retraction (ΔU1-GALL) was significantly correlated with esthetic change in the facial profile. The mean value of maxillary anterior teeth retraction was 4.88 mm, and the facial profile was significantly improved after camouflage extraction treatment (the VAS score increased from 65.89 to 77.60). The increase in nasolabial angle followed by extraction of maxillary anterior teeth had a positive influence on facial profile esthetics, indicating that a relatively obtuse nasolabial angle was acceptable in skeletal Class II patients. Waldman et al. also reported that the nasolabial angle of Class II patients improved after maxillary premolar extraction and reported a ratio of 1:3.8 between upper lip retraction and maxillary incisor retraction [25]. The retraction of anterior teeth in the adolescent group (ΔU1-GALL = 4.61 ± 1.21 mm) was lower than that in the adult group (ΔU1-GALL = 5.12 ± 1.39 mm), probably due to less use of mini-screws in adolescent patients.

It is commonly known that changes in the lower lip after anterior tooth retraction is highly predictable compared to changes in the upper lip [26]. In this study, we first found that the position of the lower lip (lower lip to E plane) was the key factor correlated with the profile esthetics of skeletal Class II patients, and improving the protrusive lower lip can achieve a pleasing facial profile. Lower lip protrusion has previously been reported to mainly depend on the maxillary incisor position instead of the mandibular incisor position [27, 28]. Indeed, the change in lower lip to the E plane and U1-GALL in our study were both found to have critical influences on subjective evaluation of facial profile esthetics during camouflage extraction treatment. Interestingly, maxillary incisor retraction (ΔU1-GALL) was significantly correlated with changes in VAS score in adult patients but failed to be significantly correlated in adolescents. There was a strong and significant correlation between the retraction of lower incisors (ΔL1-APo distance) and the increase in VAS scores in adolescent patients. These results might suggest that the retraction of lower incisors in adolescent patients and upper incisors in adult patients are important in improving facial profile during camouflage extraction treatment.

Another reason for facial profile improvement in adolescent patients was nose and mandibular growth during treatment. The nose prominence and SNB angle both showed a mild positive correlation with the change in VAS score (r = 0.135 and r = 0.129, separately). The lower facial height significantly increased and showed a negative correlation with the change in VAS score (r = − 0.261). Hence, it is important to note that the sagittal growth of the mandible is favorable for improving facial profile esthetics of skeletal Class II adolescent patients, while vertical growth of the mandible is unfavorable. The change in MP-SN angle and lower facial height in adult patients also showed negative correlations with the change in VAS score, indicating that an increase of lower facial height in skeletal Class II extraction patient could lead to an undesirable profile. Our results were consistent with the results of Shoukat Ali et al., which concluded that lower facial height significantly influences facial attractiveness, and an increase in lower facial height is considered less attractive [29]. Hence, vertical control should be considered when treating skeletal Class II extraction patients. It should be also mentioned that the facial profile of adolescent still changes after orthodontic treatment due to growth. Zierhut et al. reported progressive flattening of the facial profile could occur in adolescent after orthodontic extraction treatment, which was associated with the nose and chin growth [30]. Hence, excess retraction of anterior teeth is not recommended for adolescent. Our prediction model could reflect the posttreatment facial profile esthetics; however, the long-term profile esthetic change should be further considered for adolescent.

For skeletal Class II patients, the standard cephalometric norms should not be selected as the treatment goal. In this study, the retraction of maxillary central incisor (ΔU1-GALL) and lower lip (Δ lower lip to E plane), the increase of nasolabial angle and decrease of lower facial height, were positively correlated with the subjective assessment of profile esthetics, indicating that the camouflage treatment goal should be considered in skeletal Class II patients. Hence, the first hypothesis, that there was a strong correlation between some objective cephalometric measurements and subjective assessment of profile esthetics in skeletal Class II extraction patients, was accepted.

Artificial intelligence algorithms are widely used in orthodontic field for diagnosis and prediction, which can assist orthodontists in treatment planning [31, 32]. At present, it has been reported to identify cephalometric landmarks, detect periodontal disease, diagnose dentoskeletal classification and establish treatment plan [33, 34]. Xie et al. constructed an artificial neural network, with 80% accuracy, to determine whether premolar extraction is needed during the orthodontic treatment [35]. For mild and moderate skeletal Class II patients, orthodontists and patients are faced with the dilemma of whether to perform camouflage extraction treatment or orthodontic-orthognathic treatment. The individual prediction of posttreatment facial profile esthetics after camouflage extraction is essential for skeletal Class II patients. The prediction model could help patients decide whether the camouflage extraction treatment will satisfy their esthetic expectations and help orthodontists optimize a treatment plan. For those predicted to have an undesirable posttreatment facial profile, orthodontic-orthognathic treatment is preferred over camouflage extraction treatment. At present, prediction of posttreatment facial profile esthetics is mainly performed for orthognathic surgery patients [36–38]. To the best of our knowledge, this is the first study to predict the posttreatment facial profile esthetics of skeletal Class II extraction patients using a machine-learning method.

Our predictive model was based on patient age; 16 pretreatment measurements, which were highly and significantly correlated with facial profile esthetics according to Pearson correlation results; and the designed incisor position, ΔU1-SN, ΔL1-MP, and ΔU1-GALL, which could be planned before treatment. Comparisons of different machine-learning methods indicated that the accuracy and fitting effect of RF was superior to those of other models. The mean absolute error of RF was 3.106, which could preliminarily assist orthodontists and patients in treatment planning. In RF prediction model, all input variables contributed to the output variable. Among these, pretreatment upper lip protrusion (upper lip to E plane), pretreatment chin morphology (Pog-NB) and upper incisor retraction (ΔU1-GAll) contributed the most to the prediction model, indicating the importance of these aspects when treating skeletal Class II extraction patients. For a skeletal Class II patient with a protruded upper lip and a prominent chin, camouflage extraction treatment with upper incisor retraction could achieve a pleasing facial profile. In addition, age also played an important role in the prediction model. Hence, the treatment plan might be different between adult and adolescent patients. Based on our pilot results, the prediction of posttreatment facial profile esthetics using the RF algorithm was practical and accurate (Fig. 3). Thus, the second hypothesis of this study was also accepted.

Considering that changes in soft tissue have low predictability, the main limitation of this study was the small sample size. Further study with a larger sample size should be performed to validate our results and construct a prediction model with better performance. Besides, the facial profile esthetics is highly influenced by race. Nongthombam et al. have reported the difference of facial profile preference in different ethnicity [39]. Hence, our results were limited to Mongolian race. The evaluation and prediction of the facial profile esthetics in different human races should be further analyzed.

## Conclusions

- The lower lip protrusion is strongly correlated with subjective assessment of facial profile esthetics in skeletal Class II patients.
- Retraction of maxillary incisor and reduction of the lower lip protrusion are essential in improving the facial profile of skeletal Class II patients.
- An artificial intelligence prediction model could be a new method for predicting posttreatment facial profile esthetics. The pretreatment upper lip protrusion (upper lip to E plane), pretreatment chin morphology (Pog-NB) and upper incisor retraction (ΔU1-GAll) were the most important variables in predicting scores for facial profile esthetics.

Therefore, both null hypotheses of this study were accepted.

### Electronic supplementary material

Below is the link to the electronic supplementary material.

Supplementary Material 1

Supplementary Material 2

Supplementary Material 3

Supplementary Material 4

## Data Availability

All data analyzed during the current study are included in this published article. Any other request about the data, contact e-mail: ynsun2012@126.com.
