# Supplementary material for: Facial profile evaluation and prediction of skeletal class II patients during camouflage extraction treatment: a pilot study
Source: Head Face Med. 2023 Dec 4;19:51. doi: 10.1186/s13005-023-00397-8 (PMC10694895; doi:10.1186/s13005-023-00397-8)
Supplement: Supplementary file 1 — Supplementary Material 1 [file 13005_2023_397_MOESM1_ESM.docx]

Supplementary Table 1. Definitions of the measurements in the study

| Measurement | Definition |
| --- | --- |
| ANB (º) | The angle between the Nasion-A line and A-B line |
| SNA (º) | The angle between the Sella-Nasion line and Nasion-A line |
| SNB (º) | The angle between the Sella-Nasion line and Nasion-B line |
| Wits Appraisal (mm) | The distance between perpendiculars drawn from occlusal plane to A and B point |
| MP-SN (º) | The angle between the Sella-Nasion line and Pogonion-Menton line |
| Ar-Go-Me (º) | The angle between the Articulare-Gonion line and Gonion- Menton line |
| Y Axis (º) | The angle between the Frankfort plane and Sella-Gnathion line |
| Lower facial height (%) | The ratio of ANS-Menton to Nasion-Menton |
| Pog-NB (mm) | The distance from the Pogonion to Nasion-B line |
| Z Angle (º) | The angle between the Frankfort plane and the line through the Pogonion and the most prominent point of lower or upper lip |
| Lower Lip to E Plane (mm) | The distance from the lower lip to the line through the soft tissue Pogonion and the tip of nose |
| Upper Lip to E Plane (mm) | The distance from the upper lip to the line through the soft tissue Pogonion and the tip of nose |
| Nose Prominence (º) | The angle between the line through soft tissue Nasion and the tip of nose and the line through the tip of nose and Subnasale |
| Nasolabial Angle (º) | The angle between the line through Subnasale and upper lip and the line through Columella and Subnasale |
| Mentolabial Angle (º) | The angle between the line through soft tissue B and lower lip and the line through soft tissue B and soft tissue Pogonion point |
| U1-SN (º) | The angle between upper incisor axis and Sella-Nasion line |
| U1-APo (º) | The angle between upper incisor axis and Nasion-A line |
| U1-APo (mm) | The distance from upper incisor edge to Nasion-A line |
| L1-MP (º) | The angle between lower incisor axis and Pogonion-Menton line |
| L1-APo (º) | The angle between lower incisor axis and Nasion-A line |
| L1-APo (mm) | The distance from lower incisor edge to Nasion-A line |
| U1-L1 (º) | The angle between upper incisor axis and lower incisor axis |
| Occlusal Plane to SN (º) | The angle between the Sella-Nasion line and the occlusal plane |
| U1-GALL (mm) | The distance from upper incisor edge to the vertical reference line through the soft tissue Glabella point |
| FA-GALL (mm) | The distance from the facial-axis point of upper incisor to the vertical reference line through the soft tissue Glabella point |
