# Supplementary material for: Facial profile evaluation and prediction of skeletal class II patients during camouflage extraction treatment: a pilot study
Source: Head Face Med. 2023 Dec 4;19:51. doi: 10.1186/s13005-023-00397-8 (PMC10694895; doi:10.1186/s13005-023-00397-8)
Supplement: Supplementary file 2 — Supplementary Material 2 [file 13005_2023_397_MOESM2_ESM.docx]

**Supplementary Table 2.** Pretreatment and posttreatment subjective VAS scores and objective measurements of skeletal Class II adult and adolescent patients

| Variable | Adult group  （n=67） | | | | |  | Adolescent Group  （n=57） | | | | |  | *P* |
| --- | --- | --- | --- | --- | --- | --- | --- | --- | --- | --- | --- | --- | --- |
|  | T0 | | T1 | |  |  | T0 | | T1 | |  |  |  |
|  | mean | SD | mean | SD | *P* |  | mean | SD | mean | SD | *P* |  |  |
| **VAS score** | 64.618 | 10.628 | 77.497 | 5.105 | <0.001^**^ |  | 67.375 | 9.282 | 77.716 | 5.412 | <0.001^**^ |  | 0.129 |
| **Skeletal measurements** |  |  |  |  |  |  |  |  |  |  |  |  |  |
| ANB (º) | 6.818 | 1.380 | 5.812 | 1.284 | <0.001^**^ |  | 6.456 | 1.035 | 4.802 | 1.462 | <0.001^**^ |  | <0.001^**^ |
| SNA (º) | 83.899 | 3.764 | 82.619 | 3.506 | <0.001^**^ |  | 83.668 | 3.577 | 82.454 | 3.543 | <0.001^**^ |  | 0.678 |
| SNB (º) | 77.085 | 3.566 | 76.810 | 3.519 | <0.001^**^ |  | 77.212 | 3.208 | 77.642 | 3.285 | 0.001^*^ |  | <0.001^**^ |
| Wits Appraisal (mm) | 3.831 | 2.189 | 0.882 | 1.784 | <0.001^**^ |  | 3.847 | 1.936 | 0.437 | 1.775 | <0.001^**^ |  | 0.136 |
| MP-SN (º) | 39.127 | 5.862 | 39.396 | 5.708 | 0.017^*^ |  | 38.077 | 5.084 | 38.612 | 5.380 | 0.013^*^ |  | 0.262 |
| Ar-Go-Me (º) | 123.793 | 6.730 | 124.116 | 6.423 | 0.002^*^ |  | 124.249 | 7.084 | 124.828 | 7.407 | 0.009^*^ |  | 0.283 |
| Y Axis (º) | 73.978 | 3.673 | 74.178 | 3.649 | 0.006^*^ |  | 72.677 | 3.071 | 72.604 | 3.155 | 0.567 |  | 0.065 |
| Lower facial height (%) | 56.390 | 1.777 | 56.557 | 1.683 | 0.014^*^ |  | 55.002 | 1.724 | 55.525 | 1.922 | <0.001^**^ |  | <0.001^**^ |
| Pog-NB (mm) | -0.684 | 1.959 | -0.370 | 1.928 | 0.001^*^ |  | 0.011 | 1.379 | 0.496 | 1.437 | <0.001^**^ |  | 0.230 |
| **Soft tissue measurements** |  |  |  |  |  |  |  |  |  |  |  |  |  |
| Z Angle (º) | 59.769 | 8.422 | 68.564 | 6.458 | <0.001^**^ |  | 60.200 | 6.617 | 70.395 | 10.787 | <0.001^**^ |  | 0.390 |
| Lower Lip to E Plane (mm) | 4.442 | 2.706 | 0.693 | 1.763 | <0.001^**^ |  | 4.128 | 2.091 | 0.974 | 1.911 | <0.001^**^ |  | 0.115 |
| Upper Lip to E Plane (mm) | 0.591 | 2.120 | -0.925 | 1.763 | <0.001^**^ |  | 0.812 | 1.780 | -1.060 | 1.882 | <0.001^**^ |  | 0.162 |
| Nose Prominence (º) | 18.334 | 2.061 | 18.239 | 1.993 | 0.413 |  | 18.721 | 1.991 | 19.339 | 2.282 | 0.001^*^ |  | <0.001^**^ |
| Nasolabial Angle (º) | 105.404 | 9.556 | 112.010 | 10.095 | <0.001^**^ |  | 105.696 | 12.138 | 107.795 | 10.868 | 0.036^*^ |  | 0.001^*^ |
| Mentolabial Angle (º) | 132.918 | 40.800 | 140.758 | 13.220 | 0.107 |  | 122.505 | 43.201 | 134.002 | 14.373 | 0.055 |  | 0.627 |
|  |  |  |  |  |  |  |  |  |  |  |  |  |  |
| **Dental measurements** |  |  |  |  |  |  |  |  |  |  |  |  |  |
| U1-SN (º) | 104.407 | 8.638 | 94.230 | 6.543 | <0.001^**^ |  | 107.344 | 7.774 | 99.696 | 5.617 | <0.001^**^ |  | 0.040^*^ |
| U1-APo (º) | 36.200 | 7.824 | 24.800 | 5.162 | <0.001^**^ |  | 37.928 | 7.496 | 27.198 | 4.599 | <0.001^**^ |  | 0.589 |
| U1-APo (mm) | 9.748 | 2.399 | 5.101 | 1.476 | <0.001^**^ |  | 9.614 | 2.270 | 5.670 | 1.563 | <0.001^**^ |  | 0.061 |
| L1-MP (º) | 97.342 | 7.471 | 91.500 | 6.814 | <0.001^**^ |  | 96.225 | 6.192 | 91.904 | 5.693 | <0.001^**^ |  | 0.130 |
| L1-APo (º) | 24.681 | 5.870 | 20.333 | 4.507 | <0.001^**^ |  | 23.714 | 5.164 | 23.011 | 4.274 | 0.314 |  | 0.001^*^ |
| L1-APo (mm) | 4.869 | 2.533 | 2.093 | 1.498 | <0.001^**^ |  | 4.305 | 2.318 | 2.809 | 1.645 | <0.001^**^ |  | <0.001^**^ |
| U1-L1 (º) | 119.101 | 11.275 | 134.866 | 7.198 | <0.001^**^ |  | 118.360 | 11.109 | 129.786 | 7.451 | <0.001^**^ |  | 0.019^*^ |
| Occlusal Plane to SN (º) | 18.536 | 4.333 | -3.407 | 2.468 | <0.001^**^ |  | 17.602 | 3.606 | -2.179 | 2.348 | <0.001^**^ |  | 0.006^*^ |
| U1-GALL (mm) | 3.238 | 3.341 | -1.881 | 3.378 | <0.001^**^ |  | 0.643 | 3.553 | -3.966 | 3.005 | <0.001^**^ |  | 0.315 |
| FA-GALL (mm) | 3.737 | 3.022 | -0.798 | 2.888 | <0.001^**^ |  | 0.928 | 3.134 | -2.788 | 3.066 | <0.001^**^ |  | 0.086 |

^*^*P*<0.05, ^**^*P*<0.001
