# Supplementary material for: Facial profile evaluation and prediction of skeletal class II patients during camouflage extraction treatment: a pilot study
Source: Head Face Med. 2023 Dec 4;19:51. doi: 10.1186/s13005-023-00397-8 (PMC10694895; doi:10.1186/s13005-023-00397-8)
Supplement: Supplementary file 3 — Supplementary Material 3 [file 13005_2023_397_MOESM3_ESM.docx]

| Adult Group (n=67) | | | |  | Adolescent Group (n=57) | | | |
| --- | --- | --- | --- | --- | --- | --- | --- | --- |
| Variable | r | *P* | order |  | Variable | r | *P* | order |
| Lower Lip to E Plane (mm) | -0.640 | <0.001^**^ | 1 |  | L1-APo (mm) | -0.478 | <0.001^**^ | 1 |
| Upper Lip to E Plane (mm) | -0.593 | <0.001^**^ | 2 |  | Lower Lip to E Plane (mm) | -0.473 | <0.001^**^ | 2 |
| U1-APo (mm) | -0.505 | <0.001^**^ | 3 |  | L1-MP (º) | -0.467 | <0.001^**^ | 3 |
| Z Angle (º) | 0.493 | <0.001^**^ | 4 |  | L1-APo (º) | -0.443 | 0.001^*^ | 4 |
| U1-APo (º) | -0.487 | <0.001^**^ | 5 |  | U1-APo (mm) | -0.440 | 0.001^*^ | 5 |
| Pog-NB (mm) | 0.481 | <0.001^**^ | 6 |  | U1-L1 (º) | 0.432 | 0.001^*^ | 6 |
| U1-GALL (mm) | -0.478 | <0.001^**^ | 7 |  | Z Angle (º) | 0.365 | 0.005^*^ | 7 |
| FA-GALL (mm) | -0.470 | <0.001^**^ | 8 |  | Nasolabial Angle (º) | 0.362 | 0.006^*^ | 8 |
| L1-APo (mm) | -0.457 | <0.001^**^ | 9 |  | Upper Lip to E Plane (mm) | -0.339 | 0.010^*^ | 9 |
| U1-L1 (º) | 0.415 | 0.001^*^ | 10 |  | U1-APo (º) | -0.337 | 0.010^*^ | 10 |
| ANB (º) | -0.346 | 0.004^*^ | 11 |  | Wits Appraisal (mm) | -0.257 | 0.054 | 11 |
| U1-SN (º) | -0.311 | 0.011^*^ | 12 |  | ANB (º) | -0.215 | 0.108 | 12 |
| SNA (º) | -0.250 | 0.041^*^ | 13 |  | U1-SN (º) | -0.212 | 0.113 | 13 |
| L1-MP (º) | -0.247 | 0.044^*^ | 14 |  | Pog-NB (mm) | 0.210 | 0.117 | 14 |
| Wits Appraisal (mm) | -0.147 | 0.236 | 15 |  | Ar-Go-Me (º) | -0.191 | 0.155 | 15 |
| L1-APo (º) | -0.147 | 0.234 | 16 |  | MP-SN (º) | -0.130 | 0.334 | 16 |
| Lower facial height (%) | -0.133 | 0.282 | 17 |  | U1-GALL (mm) | -0.112 | 0.407 | 17 |
| SNB (º) | -0.131 | 0.291 | 18 |  | SNB (º) | 0.089 | 0.511 | 18 |
| Mentolabial Angle (º) | 0.121 | 0.330 | 19 |  | Nose Prominence (º) | -0.076 | 0.574 | 19 |
| Y Axis (º) | -0.041 | 0.745 | 20 |  | Y Axis (º) | -0.068 | 0.615 | 20 |
| MP-SN (º) | -0.037 | 0.764 | 21 |  | FA-GALL (mm) | -0.059 | 0.665 | 21 |
| Occlusal Plane to SN (º) | 0.016 | 0.899 | 22 |  | Lower facial height (%) | 0.054 | 0.671 | 22 |
| Nasolabial Angle (º) | 0.014 | 0.910 | 23 |  | Mentolabial Angle (º) | 0.048 | 0.724 | 23 |
| Nose Prominence (º) | 0.012 | 0.926 | 24 |  | SNA (º) | 0.018 | 0.893 | 24 |
| Ar-Go-Me (º) | 0.011 | 0.929 | 25 |  | Occlusal Plane to SN (º) | -0.001 | 0.996 | 25 |

**Supplementary Table 3.** Pearson correlation between pretreatment subjective VAS scores and objective measurements in skeletal Class II adult and adolescent patients

^*^*P*<0.05, ^**^*P*<0.001
