# Supplementary material for: Facial profile evaluation and prediction of skeletal class II patients during camouflage extraction treatment: a pilot study
Source: Head Face Med. 2023 Dec 4;19:51. doi: 10.1186/s13005-023-00397-8 (PMC10694895; doi:10.1186/s13005-023-00397-8)
Supplement: Supplementary file 4 — Supplementary Material 4 [file 13005_2023_397_MOESM4_ESM.docx]

| Adult Group (n=67) | | | |  | Adolescent Group (n=57) | | | |
| --- | --- | --- | --- | --- | --- | --- | --- | --- |
| Variable | r | *P* | order |  | Variable | r | *P* | order |
| Lower Lip to E Plane (mm) | -0.611 | <0.001^**^ | 1 |  | L1-APo (mm) | -0.461 | <0.001^**^ | 1 |
| Z Angle (º) | 0.549 | <0.001^**^ | 2 |  | Lower Lip to E Plane (mm) | -0.435 | 0.001^*^ | 2 |
| FA-GALL (mm) | -0.533 | <0.001^**^ | 3 |  | U1-L1 (º) | 0.428 | 0.001^*^ | 3 |
| U1-APo (mm) | -0.492 | <0.001^**^ | 4 |  | U1-APo (mm) | -0.406 | 0.002^*^ | 4 |
| U1-L1 (º) | 0.431 | <0.001^**^ | 5 |  | L1-APo (º) | -0.395 | 0.002^*^ | 5 |
| L1-APo (mm) | -0.421 | <0.001^**^ | 6 |  | L1-MP (º) | -0.390 | 0.003^*^ | 6 |
| U1-APo (º) | -0.399 | 0.001^*^ | 7 |  | Z Angle (º) | 0.351 | 0.008^*^ | 7 |
| U1-GALL (mm) | -0.373 | 0.002^*^ | 8 |  | U1-APo (º) | -0.313 | 0.018^*^ | 8 |
| L1-MP (º) | -0.355 | 0.003^*^ | 9 |  | U1-SN (º) | -0.289 | 0.029^*^ | 9 |
| Upper Lip to E Plane (mm) | -0.344 | 0.004^*^ | 10 |  | Lower facial height (%) | -0.261 | 0.050 | 10 |
| U1-SN (º) | -0.331 | 0.006^*^ | 11 |  | MP-SN (º) | -0.184 | 0.171 | 11 |
| ANB (º) | -0.292 | 0.016^*^ | 12 |  | Upper Lip to E Plane (mm) | -0.177 | 0.187 | 12 |
| L1-APo (º) | -0.289 | 0.018^*^ | 13 |  | Ar-Go-Me (º) | -0.165 | 0.221 | 13 |
| SNA (º) | -0.252 | 0.040^*^ | 14 |  | Nasolabial Angle (º) | 0.147 | 0.178 | 14 |
| Pog-NB (mm) | 0.181 | 0.142 | 15 |  | Nose Prominence (º) | 0.145 | 0.280 | 15 |
| Mentolabial Angle (º) | 0.171 | 0.165 | 16 |  | U1-GALL (mm) | -0.134 | 0.319 | 16 |
| Nasolabial Angle (º) | 0.167 | 0.178 | 17 |  | Y Axis (º) | -0.132 | 0.329 | 17 |
| Ar-Go-Me (º) | -0.153 | 0.215 | 18 |  | SNB (º) | 0.129 | 0.338 | 18 |
| Y Axis (º) | -0.147 | 0.237 | 19 |  | ANB (º) | -0.112 | 0.405 | 19 |
| MP-SN (º) | -0.142 | 0.250 | 20 |  | FA-GALL (mm) | 0.103 | 0.447 | 20 |
| Lower facial height (%) | -0.118 | 0.340 | 21 |  | Occlusal Plane to SN (º) | -0.102 | 0.450 | 21 |
| Wits Appraisal (mm) | -0.117 | 0.345 | 22 |  | Mentolabial Angle (º) | -0.055 | 0.685 | 22 |
| Occlusal Plane to SN (º) | -0.057 | 0.648 | 23 |  | Wits Appraisal (mm) | -0.053 | 0.696 | 23 |
| Nose Prominence (º) | -0.039 | 0.757 | 24 |  | Pog-NB (mm) | -0.031 | 0.818 | 24 |
| SNB (º) | 0.020 | 0.871 | 25 |  | SNA (º) | 0.004 | 0.978 | 25 |

**Supplementary Table 4.** Pearson correlation between subjective VAS score change and objective measurement change in skeletal Class II adult and adolescent extraction patients

^*^*P*<0.05, ^**^*P*<0.001
